# Temporal Trends and Epidemiological Patterns of Clinically Relevant Microorganisms in a Burn Unit: An 11-Year Retrospective Study from a Tertiary Hospital in Southern Brazil

**DOI:** 10.3390/epidemiologia7050130

**Published:** 2026-09-17

**Authors:** Raquel Lima Palermo, Isabela Madeira de Castro, Lais Fernanda de Almeida Spoladori, Eliandro Reis Tavares, Marcia Regina Eches Perugini, Lucy Megumi Yamauchi, Eliana Carolina Vespero, Sueli Fumie Yamada-Ogatta

**Affiliations:** 1 Programa de Pós-Graduação em Microbiologia, Departamento de Microbiologia, Universidade Estadual de Londrina, Londrina CEP 86057-970, Paraná, Brazil; raquellima.palermo@gmail.com (R.L.P.); isabela.mcastro@uel.br (I.M.d.C.); lais.spoladori@gmail.com (L.F.d.A.S.); tavares.eliandro@uel.br (E.R.T.); lionilmy@uel.br (L.M.Y.); 2 Programa de Pós-Graduação em Fisiopatologia Clínica e Laboratorial, Departamento de Patologia, Análises Clínicas e Toxicológicas, Universidade Estadual de Londrina, Londrina CEP 86038-350, Paraná, Brazil; marciaperugini@uel.br

**Keywords:** burn patients, epidemiology, Gram-negative bacteria, Gram-positive bacteria, fungi, microbial isolates

## Abstract

Background/Objectives: Burn patients are particularly vulnerable to microbial colonization and healthcare-associated microorganisms due to extensive skin barrier disruption, prolonged hospitalization, immune imbalance, and frequent invasive procedures. This study aimed to characterize the epidemiological profile and temporal trends of clinically relevant microorganisms isolated from patients admitted to a burn unit over an 11-year period. Methods: This retrospective observational study analyzed microbiological records from burn patients at a Brazilian tertiary hospital (2014–2024), including only the first isolate per patient, clinical episode, and specimen type. Microorganisms were categorized by taxon, group, and specimen sources. Temporal trends were evaluated using regression-based count models and nonparametric tests, while dominant taxa and distribution shifts were identified via epidemiological ranking, Pareto distribution, and heatmap analyses. Results: A total of 6769 clinically relevant microbial isolates were analyzed. Gram-negative bacteria predominated (60.6%), followed by Gram-positive bacteria (28.9%) and fungi (10.5%). The most frequently identified taxa were *Acinetobacter* spp. (17.3%), coagulase-negative staphylococci (13.7%), and *Pseudomonas* spp. (12.6%). Eight taxa accounted for 79.5% of all isolates, demonstrating a highly concentrated microbiological profile. Temporal analyses revealed a significant increase in Gram-positive bacterial isolates and a decline in fungal isolates throughout the study period. The distribution of microorganisms varied significantly according to clinical specimen type. Conclusions: The burn unit microbiological profile was dominated by Gram-negative pathogens and exhibited important temporal shifts over the 11-year period. These findings highlight the value of continuous local surveillance to inform infection prevention measures, and support institution-specific antimicrobial stewardship programs.

## 1. Introduction

Burn units are high-risk environments for healthcare-associated infections (HAIs), as inpatients frequently require invasive support, repeated surgical interventions, and prolonged hospitalization [1,2]. In this high-complexity setting, microbiological surveillance is a fundamental component of infection prevention and control. Therefore, the identification of predominant microorganisms and shifts in microbial profiles over time provide essential epidemiological evidence to guide empirical antimicrobial therapy and support outbreak investigations [3,4].

Furthermore, longitudinal surveillance data are critical for evaluating the effectiveness of institutional infection prevention practices. These data inform clinical decision-making, optimize patient management, and drive targeted interventions, such as patient isolation, environmental decontamination, and hand hygiene compliance, ultimately improving healthcare quality and reducing the burden of HAIs among burn patients [5,6,7].

Burn patients are particularly susceptible to infections due to disruption of the skin barrier, immune dysregulation, and frequent exposure to invasive devices such as mechanical ventilation and central venous catheters [1,8,9,10]. These factors facilitate colonization and infection by opportunistic pathogens. Evidence from the literature has highlighted temporal shifts in the microbiological epidemiology of burn patients. Gram-positive organisms are more commonly isolated during the early post-burn period, whereas Gram-negative bacteria and fungi become increasingly prevalent with prolonged hospitalization [9,10,11,12,13]. Among the most clinically relevant pathogens are *Staphylococcus aureus*, *Pseudomonas aeruginosa*, *Acinetobacter baumannii*, Enterobacterales, and fungal species of the genus *Candida* [10,12,14,15,16,17,18], which remain leading causes of morbidity and mortality in this population [2,8,9,10,17].

Studies from diverse geographic regions [19,20,21,22], including Brazil [23,24,25], have consistently reported a predominance of Gram-negative organisms in burn units, with *Acinetobacter* spp., *Pseudomonas* spp., and *Klebsiella* spp. among the most frequently reported taxa. Nevertheless, the epidemiology of infections in burn units remains highly dynamic [12,13,14,26,27,28,29], varying according to geographic region, local infection control practices, antimicrobial consumption, length of hospital stay, and environmental factors [10]. This heterogeneity underscores the limited external validity of data derived from individual centers and reinforces the need for continuous local surveillance to characterize pathogen distribution and temporal trends. Establishing a local epidemiological baseline is essential for guiding empirical antimicrobial therapy, monitoring changes in microbial ecology over time, and supporting the evaluation of infection prevention and control practices [14].

Despite numerous reports describing microorganisms in burn units, few studies have evaluated long-term temporal trends over more than one decade using standardized epidemiological methods in Latin America. Consequently, long-term changes in microbial ecology and pathogen distribution remain poorly characterized in Brazilian burn centers, particularly in referral burn units, where continuous epidemiological surveillance is essential for guiding infection prevention and antimicrobial stewardship programs [30].

Therefore, this study aimed to characterize the epidemiological profile and temporal trends of clinically relevant microorganisms isolated from patients admitted to a burn unit at a tertiary care hospital in southern Brazil over an 11-year period (2014–2024).

## 2. Materials and Methods

### 2.1. Study Design and Setting

This retrospective, observational, single-center study was conducted at a tertiary care hospital in southern Brazil, which serves as a regional referral center for burn care for the Sistema Único de Saúde (SUS), a governmental public health system. The burn unit comprises an inpatient ward and a dedicated burn intensive care unit (BICU), providing care for patients with moderate-to-severe burn injuries from multiple municipalities across the region. Patients were either directly admitted to the study hospital or transferred from other healthcare facilities. Among transferred patients, standardized information regarding the duration of hospitalization at the referring institution, timing of transfer, and microbiological status before admission to the burn unit was not routinely collected and was therefore unavailable for analysis. Microbiological records from burn patients admitted to the burn ward or BICU between January 2014 and December 2024 were analyzed. This study was designed as an isolate-based epidemiological investigation aimed at characterizing the distribution and temporal trends of clinically relevant microorganisms recovered from burn patients.

### 2.2. Ethical Issue

This study was conducted in accordance with the ethical principles governing research involving human subjects and was approved by the Research Ethics Committee of the Universidade Estadual de Londrina [UEL; Certificado de Apresentação de Apreciação Ética (CAAE) 80467624.2.0000.5231, and opinion number 7.118.248]. As this was a retrospective cross-sectional study based on data and biological samples obtained from routine hospital diagnostic procedures, a waiver of informed consent was requested. The samples analyzed had been collected from patients treated during different time periods, making individual identification and contact unfeasible. All data were analyzed in an anonymized manner, with access restricted to the research team, ensuring the confidentiality and privacy of participant information. The results are presented in aggregate form, precluding the identification of individual participants.

### 2.3. Data Collection

Microbiological data were retrieved from the hospital LABHOS (Laboratório Hospitalar) management system. For the purposes of this retrospective study, a clinical episode was defined as a distinct clinical event that prompted a microbiological investigation, with the distinction between episodes determined based on the available laboratory records and the clinical context associated with specimen collection. To avoid overrepresentation, only the first isolate per patient, clinical episode, and specimen type was included. Surveillance cultures and duplicate isolates of the same taxon recovered from the same specimen type within the same clinical episode were excluded, with the first isolate retained. Isolates representing different taxa or recovered from different specimen types were considered distinct microbiological events. Therefore, a subsequent clinical episode could contribute an additional isolate from the same patient.

The microbiological dataset consisted of clinical culture records generated during routine clinical care from specimens obtained from patients hospitalized in the burn ward or BICU. Specimens were collected according to the clinician’s assessment and clinical indication from a variety of specimens, including blood, urine, tracheal aspirates, wound exudates, biological fluids, tissue specimens (including skin fragments, soft tissue specimens, and debridement tissue), and other clinical materials.

Therefore, the denominator for the descriptive and temporal analyses was the total number of eligible microbial isolates/records, and not the total number of cultures performed or the total number of hospitalized patients. After the sequential data-cleaning process, 6769 microbiological records were included in the final analytical dataset.

Culture results were initially classified as positive or negative. Microorganisms were classified by microbiological group [Gram-negative bacteria (GNB), Gram-positive bacteria (GPB), or fungi], taxon, and clinical specimen source.

### 2.4. Microbial Identification

Microorganism identification was performed according to the routine procedures of the hospital microbiology laboratory, using the manual and automated methods available during the study period. Microorganisms recovered from clinical specimens were identified using conventional phenotypic methods based on colony morphology, Gram staining, and standard biochemical tests, following the recommendations of the Clinical and Laboratory Standards Institute [31], as well as by automated identification systems (VITEK^®^ 2, bioMérieux, Marcy-l’Étoile, France, and/or BD Phoenix™, Becton Dickinson, Sparks, MD, USA), according to the manufacturers’ instructions.

### 2.5. Statistical Analysis

Descriptive statistics were used to summarize the microbiological profile of the study population. Categorical variables were presented as absolute frequencies (*n*) and relative percentages (%). The distribution of microorganisms was evaluated according to microbiological group, taxon, and clinical specimen type.

Annual temporal trends in microbiological culture results were assessed using Kendall’s rank correlation test. Differences in the distribution of microbiological groups across specimen types were assessed using the chi-square test or Fisher’s exact test, as appropriate.

Temporal changes in isolate counts were evaluated using count regression models with calendar year as the independent variable. Poisson regression was initially considered, and negative binomial regression was applied when overdispersion was detected. The results are presented as incidence rate ratios (IRRs) per year with 95% confidence intervals (95% CIs). For the most frequent taxa, *p*-values were adjusted for multiple comparisons using the false discovery rate (FDR) method, and an adjusted *q*-value < 0.05 was considered statistically significant.

A Pareto analysis was conducted to identify the microorganisms contributing most substantially to the cumulative microbiological burden. Heatmap visualization was used to explore temporal variations in the distribution of the most frequent taxa throughout the study period.

All statistical analyses were performed using R software (version 4.3.0) and IBM SPSS Statistics (version 26.0). Statistical significance was defined as *p* < 0.05 for inferential analyses, except where FDR-adjusted *q*-values were applied.

## 3. Results

### 3.1. Temporal Trends in Microbiological Isolates in a Burn Unit

**Figure 1:**
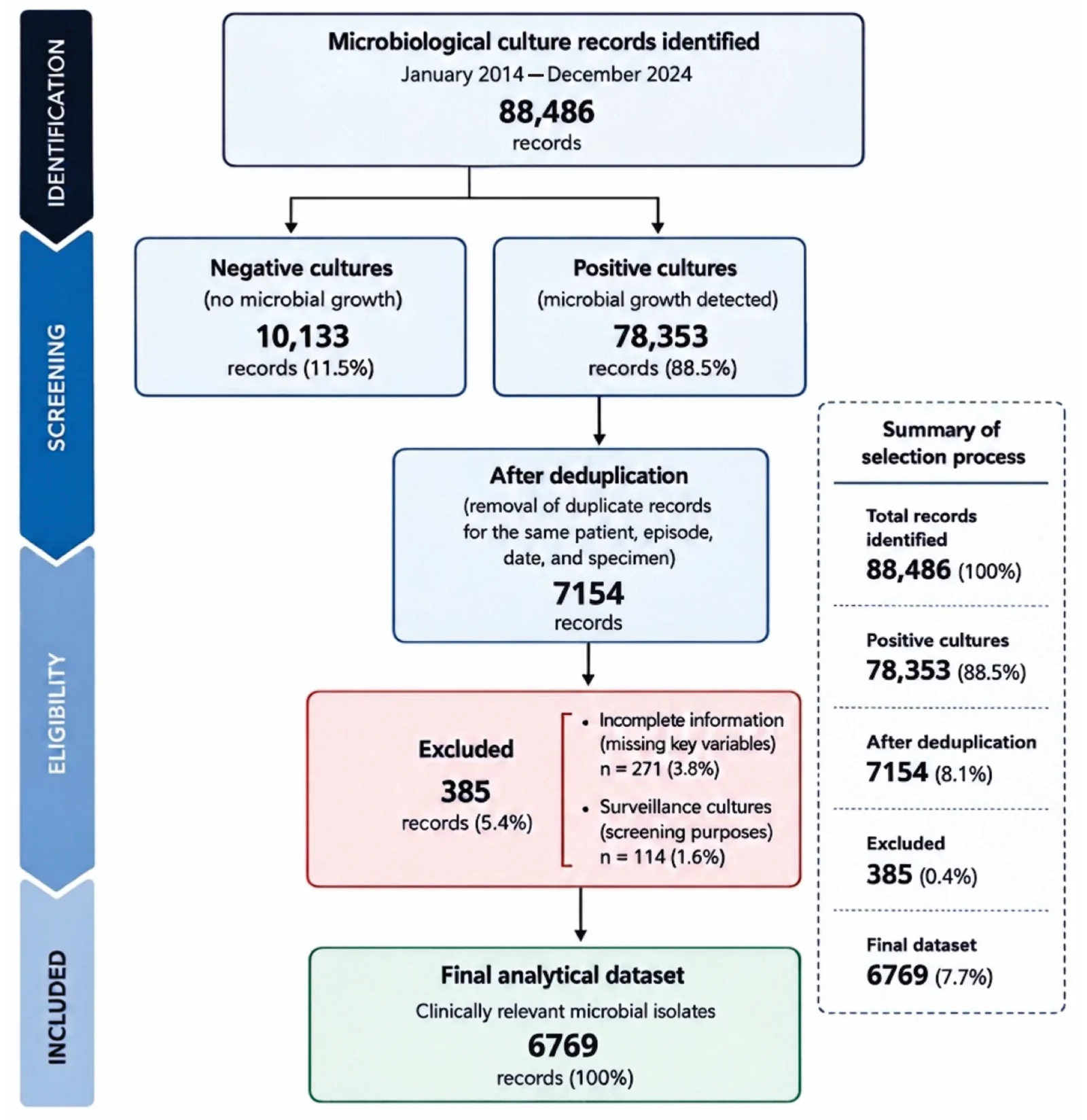
Flow diagram of microbiological record identification, screening, eligibility assessment, and inclusion in the final analytical dataset.

From 2014 to 2024, 88,486 microbiological culture records were identified, including 10,133 negative and 78,353 positive cultures. Positive cultures increased from 3486 in 2014 to 8190 in 2024, whereas negative cultures decreased from 1407 to 390, corresponding to significant temporal trends (Kendall’s τ = 0.491, *p* = 0.0405 and τ = −0.564, *p* = 0.0165, respectively). After deduplication, 7154 positive records remained. Of these, 385 cultures were subsequently excluded because of incomplete information or because they were obtained for surveillance purposes (Figure 1). The number of deduplicated positive records increased from 437 in 2014 to 751 in 2024 (Kendall’s τ = 0.527, *p* = 0.0264). The proportion of positive cultures among all culture records also increased significantly, from 71.2% in 2014 to 95.5% in 2024 (Kendall’s τ = 0.927, *p* < 0.001).

This study included 6769 microbiological records corresponding to identified microbial isolates (Figure 1), representing 61 taxonomically standardized microorganisms identified across nine distinct clinical specimen types. The annual number of isolates increased from 345 in 2014 to 744 in 2024, with a peak of 828 isolates recorded in 2023. Poisson regression analysis demonstrated a significant temporal increase in the number of isolates over the study period (IRR per year = 1.065; 95% CI: 1.038–1.094; *p* = 2.45 × 10^−6^), corresponding to an average annual increase of 6.5%.

### 3.2. Microbial Isolates Recovered from Hospitalized Burn Patients

Overall, the microbiological profile was dominated by Gram-negative bacteria, which accounted for 4103 isolates (60.6%), followed by Gram-positive bacteria (1957 isolates; 28.9%) and fungi (709 isolates; 10.5%) (Table 1).

Pareto analysis revealed a highly concentrated distribution of microorganisms, with a limited number of taxa accounting for the majority of clinical isolates. Specifically, eight taxa overall represented approximately 80% of all isolates, whereas the remaining species formed a long tail of low-frequency microorganisms (Supplementary Figure S1).

The most frequently isolated microorganisms were *Acinetobacter* spp. (1172 isolates; 17.3%), coagulase-negative staphylococci (CoNS—927; 13.7%), *Pseudomonas* spp. (856; 12.6%), *Klebsiella* spp. (727; 10.7%), *Candida* spp. (515; 7.6%), *Staphylococcus aureus* (460; 6.8%), *Enterococcus* spp. (396; 5.9%), and *Enterobacter* spp. (326; 4.8%) (Table 1).

Other opportunistic pathogens, including *Serratia* spp., *Escherichia coli*, and *Proteus* spp., were recovered at lower frequencies. Rare microorganisms, each accounting for less than 1% of isolates, included environmental and opportunistic species such as *Stenotrophomonas maltophilia*, *Burkholderia cepacia* complex, *Elizabethkingia meningoseptica*, and *Chryseobacterium* spp., underscoring the ecological diversity of the burn unit microorganisms.

### 3.3. Distribution of Microbial Isolates by Clinical Specimens

Regarding clinical specimen distribution, microbial isolates were recovered predominantly from tissue samples (1907; 28.2%), followed by catheter-derived specimens (1476; 21.8%), tracheal aspirates (1434; 21.2%), urine samples (1067; 15.8%), and blood cultures (710; 10.5%) (Table 2).

The microbiological composition differed significantly among specimen types, reflecting distinct ecological niches (χ^2^ = 1238.8, *p* < 0.001; Cramér’s V = 0.302). A moderate-to-strong association was observed between specimen source and microbial group. Overall, Gram-negative bacteria predominated in tissue, catheter-derived, tracheal aspirate, and urine samples, whereas blood cultures exhibited the highest proportional contribution of Gram-positive organisms (50.6%). Fungal isolates were disproportionately represented in urine specimens, accounting for 33.6% of isolates, primarily due to *Candida* spp. and *Trichosporon* spp.

Distinct taxonomic patterns were also observed across specimen types. *Acinetobacter* spp. was the predominant genus in tissue samples and tracheal aspirates, while CoNS were the leading isolates recovered from blood cultures and catheter-derived specimens. In urine samples, *Candida* spp. represented the most frequently isolated pathogen. These specimen-specific microbiological profiles are summarized in Table 2, and the proportional distribution of the major microbiological groups across specimen types is presented in Supplementary Figure S2.

### 3.4. Epidemiological Dynamics of Microbial Isolates in a Burn Unit

Temporal trend analysis revealed significant changes in the relative abundance of the major microbiological groups over the study period. Gram-negative bacteria remained proportionally stable (IRR/year = 0.998; *p* = 0.426), whereas Gram-positive bacteria exhibited a significant increasing trend (IRR/year = 1.028; *p* = 0.0018). In contrast, fungal isolates showed a significant decline over time (IRR/year = 0.942; *p* = 0.0027) (Table 3).

At the species level, significant temporal changes were also observed among the ten most prevalent taxa after FDR correction. *Enterobacter* spp. (IRR/year = 0.931; *q* = 1.40 × 10^−5^), *Candida* spp. (IRR/year = 0.923; *q* = 0.0084), and *Acinetobacter* spp. (IRR/year = 0.973; *q* = 0.0084) demonstrated significant decreasing trends over time. Conversely, *S. aureus* (IRR/year = 1.053; *q* = 8.05 × 10^−4^) and *Enterococcus* spp. (IRR/year = 1.076; *q* = 0.013) showed significant increases. Although *Pseudomonas* spp. exhibited an upward trend, this association did not remain statistically significant after adjustment for multiple comparisons (*q* = 0.0968) (Table 4).

**Figure 2:**
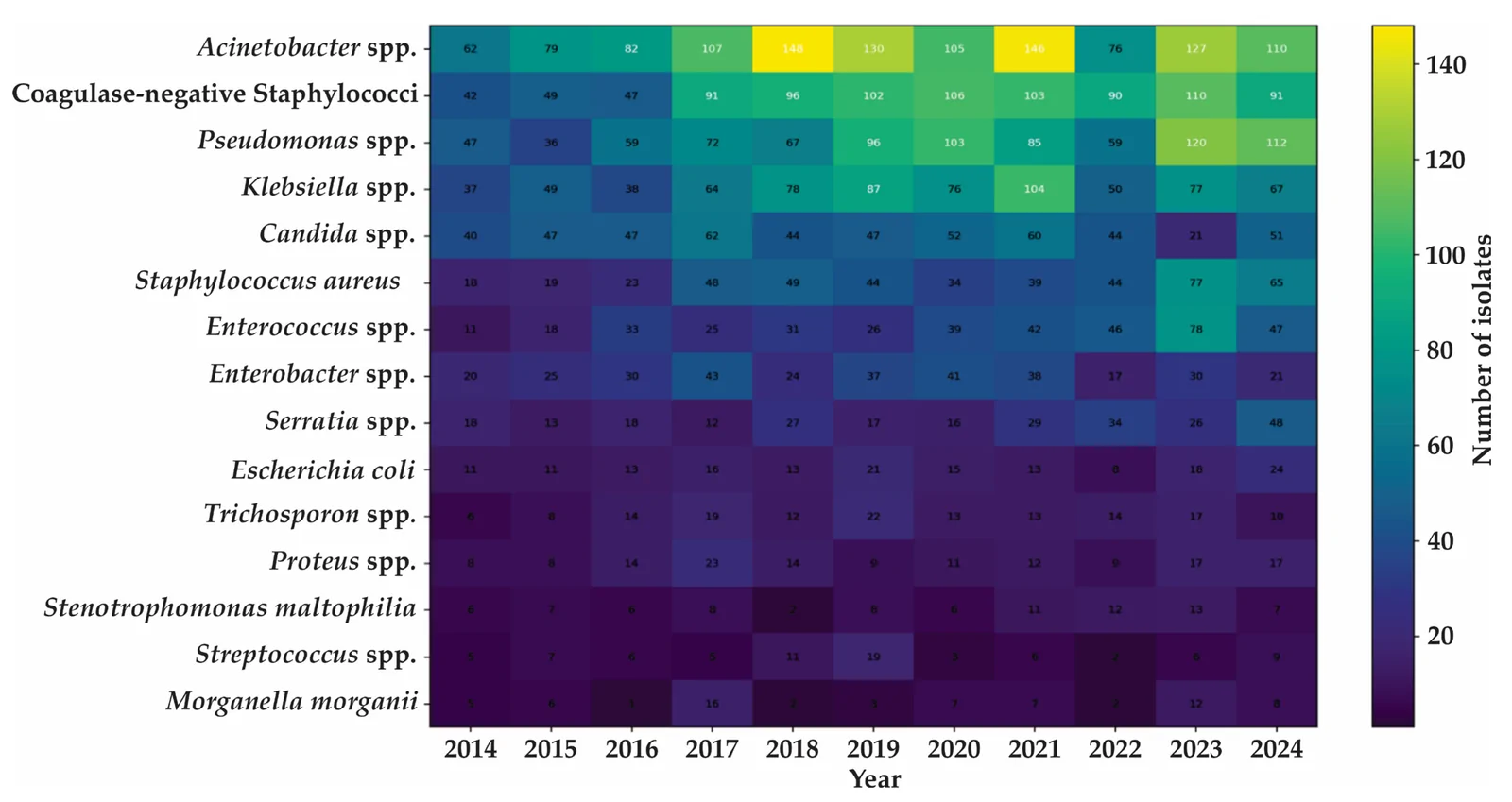
Temporal heatmap of major microbial isolates in a burn unit of a tertiary hospital of southern Brazil (2014–2024).

Temporal heatmap analysis further demonstrated heterogeneous fluctuations in the relative abundance of the most frequent taxa, indicating a dynamic and evolving microbiological landscape rather than persistent dominance of any single species. These temporal patterns are illustrated in Figure 2.

## 4. Discussion

The present study provides a longitudinal overview of the epidemiology of clinical microorganisms isolated from burn patients over an 11-year period at a tertiary hospital in southern Brazil. The observed average annual increase of 6.5% in isolate counts probably reflects a combination of factors, including rising patient admissions, increased microbiological sampling, expansion of critical care services, and improvements in laboratory diagnostic capacity, rather than a true increase in HAI incidence.

The substantial number of records excluded during deduplication highlights the importance of accounting for repeated microbiological sampling in retrospective studies of burn patients, particularly in the setting of prolonged hospitalization and repeated clinical investigations. The increase in positive records persisted after deduplication, suggesting that the temporal pattern was not solely attributable to repeated cultures from the same patients. However, these findings should be interpreted as changes in microbiological culture positivity rather than changes in infection incidence, as patient-level denominators and clinically defined infection episodes were not consistently available. The progressive increase in the proportion of positive cultures may reflect changes in patient severity, sampling practices, referral patterns, or institutional microbiological surveillance and diagnostic strategies over the 11-year period. Exclusion of surveillance cultures and records with incomplete information was therefore applied to improve the epidemiological interpretability and comparability of the final dataset.

Overall, Gram-negative bacteria, particularly non-fermenting bacilli, predominated among the microorganisms recovered from microbiological cultures of burn patients at the study hospital, followed by Gram-positive cocci and fungal pathogens. Longitudinal analysis revealed a dynamic microbiological profile. While the proportion of Gram-negative bacteria remained relatively stable throughout the study period, Gram-positive organisms increased significantly, whereas fungal isolates showed a significant decline over time.

The predominance of Gram-negative bacteria observed in this study aligns with reports from burn centers worldwide [17,18,19,20,21,22], highlighting the central role of these organisms in HAIs among burn patients. A similar taxonomic distribution was observed in a previous study conducted at the same hospital between 2009 and 2013, in which *Acinetobacter* spp., *Pseudomonas* spp., *Klebsiella* spp., and other Enterobacterales (*Enterobacter* spp., *E. coli*, and *Serratia* spp.) were identified as the leading causative agents of HAIs in burn patients. In contrast to our findings, *S. aureus* and *Enterococcus faecalis* were the predominant Gram-positive bacteria, ranking as the sixth and eighth most common pathogens overall, respectively [23].

In the present study, *Acinetobacter* spp. was the leading taxon, accounting for 17.3% of all isolates. While it remained the most frequently recovered taxon over the study period, reflecting its persistence as an endemic component of the local burn-unit ecology, its relative abundance showed a significant declining trend over time. *Pseudomonas* spp. ranked as the second most prevalent taxon, accounting for 12.6% of all isolates. Despite its consistent predominance throughout the study period, its temporal trend was no longer statistically significant after adjustment for multiple comparisons. Clinically, both non-fermenting bacilli are well-recognized causes of wound, respiratory, and device-associated infections in burn centers [2,8,9,10,16,32]. Although our analysis was based on microbiological isolates rather than clinically confirmed infections, and therefore could not distinguish colonization from true infection, both microbial taxa were predominantly recovered from tissue, tracheal aspirate and catheter-derived specimens.

*Klebsiella* spp. was the third most frequently isolated Gram-negative taxon. In addition, other members of the order Enterobacterales, including *Enterobacter* spp., *Serratia* spp., and *E. coli*, ranked among the ten most frequently isolated microorganisms. Although *Enterobacter* spp. exhibited a significant declining trend over time, both *Klebsiella* spp. and *Enterobacter* spp. remained common throughout the surveillance period. These opportunistic organisms are important causes of respiratory, urinary tract, bloodstream, and wound infections in burn patients, particularly among those requiring prolonged hospitalization or invasive procedures [2,9,10,32].

Collectively, these Gram-negative bacteria are recognized for their remarkable persistence in hospital settings, efficient colonization of compromised tissues, biofilm-forming capacity, and ability to acquire multidrug resistance [7,9,24,25,33]. Notably, carbapenem resistance and third-generation cephalosporin resistance are major determinants of their clinical and public health importance. Accordingly, the World Health Organization (WHO) has classified carbapenem-resistant *A. baumannii* and third-generation cephalosporin-resistant and carbapenem-resistant Enterobacterales as critical-priority pathogens, and carbapenem-resistant *P. aeruginosa* as a high-priority pathogen, underscoring the urgent need for new strategies to prevent and treat infections caused by these bacteria [30].

Recent Brazilian studies have likewise reported the predominance of Gram-negative bacteria in burn units. Risseto et al. [24] evaluated the microbiological profile of burn patients admitted to a university hospital in southeastern Paraná during two study periods (2015–2016 and 2019–2020) and similarly identified *P. aeruginosa*, *A. baumannii*, and *Klebsiella* spp. as the most frequently isolated microorganisms. The main difference between the two studies was the predominance of *S. aureus* as the leading Gram-positive pathogen in their cohort, whereas CoNS were more frequently isolated in our series.

Similarly, a retrospective study conducted at a university hospital in the state of São Paulo between 2018 and 2022 reported *Acinetobacter* spp., *Pseudomonas* spp., and *Klebsiella* spp. as the predominant pathogens among burn patients with HAIs. In contrast, *Enterococcus* spp. was the predominant Gram-positive genus in infection episodes in that cohort, and burn wound infections represented the most frequent infection site [25].

A notable finding of the present study was the significant increase in the relative contribution of Gram-positive bacteria over the 11-year surveillance period, despite the persistent predominance of Gram-negative organisms. This trend was primarily driven by the increasing frequency of *S. aureus* and *Enterococcus* spp., suggesting a gradual shift in the microbiological profile of burn patients. Because standardized patient-level information regarding admission volume, clinical severity, and changes in healthcare practices was not available for the entire study period, the underlying causes of the observed increase in Gram-positive bacteria could not be directly determined. However, the burn unit increasingly operated as a regional referral service and managed patients with greater clinical complexity over time, which may have contributed to the observed increase. No formal changes in the microbiological sampling protocol or in the timing of specimen collection were identified that could explain this trend. Nevertheless, differences in patient characteristics, antimicrobial selective pressure, or clinical practices may also have played a role. Furthermore, improvements in the control of traditionally dominant Gram-negative pathogens may have altered the relative distribution of bacterial isolates over time. Similar shifts toward greater participation of Gram-positive organisms have been reported in burn centers and other critical care settings, highlighting the dynamic nature of hospital microbial ecology [12,13,14,16,18,26,27,28,29].

The increasing prevalence of *S. aureus* is clinically significant because, despite being a common constituent of the human microbiota [34], it remains one of the leading causes of invasive HAIs [1,9,16,29,35]. Its pathogenicity is driven by a broad repertoire of virulence factors that promote tissue adhesion, invasion, immune evasion, and hematogenous dissemination, thereby contributing to severe invasive disease and increased mortality [36]. In burn patients, *S. aureus* is one of the predominant pathogens associated with burn wound infections and subsequent bloodstream infections [1,16].

Coagulase-negative staphylococci (CoNS) also represented a substantial proportion of isolates, particularly in blood culture and catheter-derived specimens. CoNS are common constituents of the normal skin microbiota [34] and may therefore represent either culture contamination or true infection. Nevertheless, these organisms are increasingly recognized as significant opportunistic pathogens, particularly in patients requiring prolonged intravascular device use, owing to their ability to adhere to biomaterial surfaces and form biofilms [16,35,37,38]. The predominance of CoNS in device-related specimens observed in this study therefore likely reflects the extensive use of invasive support in burn care and reinforces the importance of careful clinical interpretation of blood culture results, together with strict catheter management and infection prevention practices.

The increasing frequency of *Enterococcus* spp. is also noteworthy, given its recognized role in catheter-associated, surgical-site, urinary tract, and bloodstream infections among critically ill patients [9,25,35]. The emergence of enterococci as important nosocomial pathogens, including in burn units, has been attributed to prolonged hospitalization, exposure to broad-spectrum antimicrobial agents, and the selective pressure exerted by antimicrobial therapy, which promotes the persistence and dissemination of intrinsically resistant Gram-positive organisms [1,39].

Fungal taxa, particularly *Candida* spp. and *Trichosporon* spp., represented a consistent, albeit proportionally smaller, component of the microbiological profile throughout the surveillance period and exhibited a significant declining trend over time. The predominance of yeasts in urine specimen observed in our cohort is consistent with reports from critically ill populations, in which colonization often precedes invasive infection, particularly in the presence of biofilm-forming organisms and impaired host defenses [15,16,40]. These findings are clinically relevant in burn patients, as invasive fungal infections are typically associated with greater disease severity, prolonged hospitalization, exposure to broad-spectrum antibiotics, and the extensive use of invasive devices [15,16,40,41,42]. Importantly, the WHO has highlighted the increasing clinical relevance of fungal pathogens, particularly *Candida* and *Aspergillus* species, in the context of antimicrobial resistance and critical care infections [43].

Although the decline in fungal isolates may reflect improvements in antimicrobial stewardship, more judicious use of invasive devices, or strengthened infection prevention and control practices, this interpretation should be made cautiously, as changes in culturing practices and underdiagnosis may also have influenced fungal detection over time. Despite the declining trend observed in this study, the persistent recovery of fungal pathogens underscores the importance of continued microbiological surveillance, careful distinction between colonization and infection, and the incorporation of antifungal stewardship into infection prevention and control strategies in burn units.

The detection of less common opportunistic pathogens, including *Elizabethkingia meningoseptica*, *Chryseobacterium* spp., and *Achromobacter* spp., further illustrates the ecological complexity of the burn-unit microbiota. Notwithstanding their low frequency, these organisms are increasingly recognized as opportunistic healthcare-associated pathogens and may become clinically significant in patients with prolonged hospitalization, extensive antimicrobial exposure, and severe underlying illness or immunological vulnerability [44,45,46].

Even though the major microbiological taxa exhibited distinct temporal trends, they consistently showed recurrent year-to-year fluctuations throughout the study period. These dynamic variations likely reflect the complex interplay of changes in patient case mix and disease severity, antimicrobial selective pressure, invasive device utilization, environmental contamination, healthcare practices, and microbiological sampling intensity, although these variables were not directly assessed in the present study. Consequently, the temporal fluctuations observed should be interpreted as multifactorial ecological changes rather than evidence of any single underlying cause. From an epidemiological perspective, the finding that nearly 80% of all isolates were concentrated within a limited number of taxa indicates that the microbiological profile of the burn unit was driven by a relatively small group of clinically important pathogens. This structured distribution has direct implications for clinical practice, supporting targeted microbiological surveillance, periodic revision of unit-specific antibiograms, rigorous infection prevention measures, and antimicrobial stewardship programs directed toward the microorganisms responsible for the greatest burden of HAIs [9,35,47].

Several limitations should be acknowledged when interpreting the findings of this study. First, this study analyzed microbiological isolates rather than individual patients. Consequently, the findings reflect the microbiological burden of the burn unit rather than the incidence of clinically confirmed infections. Moreover, because of the retrospective design and the lack of detailed clinical information, it was not possible to distinguish colonization from true infection. This limitation is particularly relevant for microorganisms such as CoNS and urinary yeasts, which may represent either colonization or clinically significant infection depending on the clinical context. Nevertheless, colonization by many of the identified microorganisms is a well-recognized risk factor for subsequent infection in immunocompromised patients [40,48,49], underscoring the clinical value of microbiological surveillance. Whenever possible, microbiological findings should be interpreted in conjunction with clinical and laboratory data.

Second, the retrospective laboratory dataset did not contain standardized information regarding the duration of hospitalization at referring institutions, timing of transfer, or microbiological status before admission to our burn unit was not available for transferred patients. Consequently, we were unable to quantitatively assess the potential contribution of pre-admission colonization to the observed microbiological profile.

Another important limitation is the lack of antimicrobial susceptibility data. Because resistance phenotypes were not evaluated, it was not possible to characterize temporal trends in antimicrobial resistance or assess the prevalence of multidrug-resistant organisms. Nevertheless, many of the predominant taxa identified in this study, including *Acinetobacter* spp., *Pseudomonas* spp., members of the Enterobacterales order, *S. aureus* and *Enterococcus* spp. are globally recognized as major healthcare-associated pathogens frequently associated with multidrug resistance [29,30,35,39,49]. Integrating longitudinal microbiological surveillance with antimicrobial susceptibility data should therefore be a priority for future investigations.

Finally, this was a single-center study conducted in a tertiary referral burn unit, and the observed microbiological profile may reflect local epidemiological characteristics, antimicrobial use practices, and infection prevention policies. Consequently, caution is warranted when extrapolating these findings to other institutions or healthcare settings. Nevertheless, the extended 11-year surveillance period and the large number of microbiological isolates provide a comprehensive overview of the local microbial ecology and strengthen the internal validity of the observed temporal trends.

Overall, the present study provides one of the longest longitudinal descriptions of the microbiological ecology of a Brazilian burn unit and offers valuable epidemiological findings for guiding local surveillance, empirical antimicrobial therapy, and future investigations integrating microbiological, clinical, and antimicrobial resistance data.

## 5. Conclusions

Collectively, the findings of the present study demonstrate that the microbiological profile of the burn unit remained dynamic over the 11-year study period, with persistent predominance of Gram-negative bacteria accompanied by a gradual increase in Gram-positive organisms and a decline in fungal isolates. The concentration of nearly 80% of all isolates within a limited number of taxa further indicates that a relatively small group of pathogens accounts for most of the microbiological burden. These observations reinforce the value of long-term microbiological surveillance for detecting temporal shifts in pathogen distribution and for supporting evidence-based infection prevention strategies and empirical antimicrobial therapy in burn care.

## Figures and Tables

**Table 1:** Microbial isolates recovered from hospitalized burn patients of a tertiary hospital of southern Brazil during 2014–2024.

Rank	Microorganism ^a^	Group	*n* Isolates (%) ^b^	Cumulative %
1	*Acinetobacter* spp.	GNB	1172 (17.3)	17.3
2	Coagulase-negative staphylococci	GPB	927 (13.7)	31.0
3	*Pseudomonas* spp.	GNB	856 (12.6)	43.7
4	*Klebsiella* spp.	GNB	727 (10.7)	54.4
5	*Candida* spp.	Fungi	515 (7.6)	62.0
6	*Staphylococcus aureus*	GPB	460 (6.8)	68.8
7	*Enterococcus* spp.	GPB	396 (5.9)	74.6
8	*Enterobacter* spp	GNB	326 (4.8)	79.5
9	*Serratia* spp.	GNB	258 (3.8)	83.3
10	*Escherichia coli*	GNB	163 (2.4)	85.7
11	*Trichosporon* spp.	Fungi	148 (2.2)	87.9
12	*Proteus* spp.	GNB	142 (2.1)	90.0
13	*Stenotrophomonas maltophilia*	GNB	86 (1.3)	91.2
14	*Streptococcus* spp.	GPB	79 (1.2)	92.4
15	*Morganella morganii*	GNB	69 (1.0)	93.4
16	*Bacillus* spp.	GPB	67 (1.0)	94.4
17	*Providencia* spp.	GNB	65 (1.0)	95.4
18	*Citrobacter* spp.	GNB	44 (0.7)	96.0
19	*Burkholderia cepacia complex*	GNB	43 (0.6)	96.7
20	*Chryseobacterium* spp.	GNB	28 (0.4)	97.1
21	*Aeromonas* spp.	GNB	24 (0.4)	97.4
22	*Fusarium* spp.	Fungi	21 (0.3)	97.7
23	*Aspergillus* spp.	Fungi	18 (0.3)	98.0
24	*Elizabethkingia meningoseptica*	GNB	15 (0.2)	98.2
25	*Achromobacter* spp.	GNB	13 (0.2)	98.4
26	*Pantoea* spp.	GNB	11 (0.2)	98.6
27	*Sphingomonas paucimobilis*	GNB	10 (0.1)	98.7
28	*Cupriavidus pauculus*	GNB	9 (0.1)	98.9
29	*Brevundimonas* spp.	GNB	8 (0.1)	99.0
30	*Delftia acidovorans*	GNB	7 (0.1)	99.1
31	*Corynebacterium* spp.	BGP	6 (0.1)	99.2
32	*Lactococcus* spp.	BGP	5 (0.1)	99.2
33	*Haemophilus* spp.	BGN	4 (0.1)	99.3
34	*Pandoraea* spp.	BGN	4 (0.1)	99.4
35	*Micrococcus* spp.	BGP	4 (0.1)	99.4
36	*Moraxella* spp.	GNB	3 (<0.1)	99.5
37	*Kocuria kristinae*	GPB	3 (<0.1)	99.5
38	*Alcaligenes faecalis*	GNB	3 (<0.1)	99.6
39	*Leuconostoc* spp.	GPB	3 (<0.1)	99.6
40	*Raoultella* spp.	GNB	2 (<0.1)	99.6
41	*Kodamaea ohmeri*	Fungi	2 (<0.1)	99.7
42	*Aerococcus viridans*	GPB	2 (<0.1)	99.7
43	*Acremonium* spp.	Fungi	2 (<0.1)	99.7
44	*Oligella ureolytica*	GNB	2 (<0.1)	99.7
45	*Brevibacterium* spp.	GPB	1 (<0.1)	99.8
46	*Kluyvera intermedia*	GNB	1 (<0.1)	99.8
47	*Hafnia alvei*	GNB	1 (<0.1)	99.8
48	*Granulicatella adiacens*	GPB	1 (<0.1)	99.8
49	*Buttiauxella agrestis*	GNB	1 (<0.1)	99.8
50	*Mucor* spp.	Fungi	1 (<0.1)	99.8
51	*Lactobacillus* spp.	GPB	1 (<0.1)	99.9
52	*Leclercia adecarboxylata*	GNB	1 (<0.1)	99.9
53	*Ochrobactrum anthropic*	GNB	1 (<0.1)	99.9
54	*Paecilomyces* spp.	Fungi	1 (<0.1)	99.9
55	*Paenibacillus* spp.	GPB	1 (<0.1)	99.9
56	*Neisseria meningitidis*	GNB	1 (<0.1)	99.9
57	*Propionebacterium* spp.	GPB	1 (<0.1)	99.9
58	*Salmonella* spp.	GNB	1 (<0.1)	100
59	*Saccharomyces cerevisiae*	Fungi	1 (<0.1)	100
60	*Shewanella putrefasciens*	GNB	1 (<0.1)	100
61	*Vibrio fluvialis*	GNB	1 (<0.1)	100

^a^ Microbial genus or species was identified by phenotypic methods using conventional microbiological methods and automated identification systems (VITEK^®^ 2 and/or BD Phoenix™); ^b^ *n*: Total of isolates in each group, and percentage among all isolates. GNB: Gram-negative bacteria; GPB: Gram-positive bacteria.

**Table 2:** Distribution of microbial isolates by clinical specimen type.

Specimen	GNB	GPB	Fungi	Total (%) ^a^	Top 1 Taxon ^b^	Top 2 Taxon ^b^	Top 3 Taxon ^b^
Tissue ^c^	1265	558	84	1907 (28.2)	*Acinetobacter* spp. (*n* = 396)	*Pseudomonas* spp. (*n* = 360)	*Enterococcus* spp. (*n* = 196)
Catheter-derived	733	650	93	1476 (21.8)	CoNS (*n* = 458)	*Acinetobacter* spp. (*n* = 243)	*Klebsiella* spp. (*n* = 149)
Tracheal aspirate	1068	236	130	1434 (21.2)	*Acinetobacter* spp. (*n* = 338)	*Klebsiella* spp. (*n* = 209)	*Pseudomonas* spp. (*n* = 186)
Urine	606	102	359	1067 (15.8)	*Candida* spp. (*n* = 239)	*Klebsiella* spp. (*n* = 170)	*Trichosporon* spp. (*n* = 118)
Blood	316	359	35	710 (10.5)	CoNS (*n* = 254)	*Acinetobacter* spp. (*n* = 91)	*Klebsiella* spp. (*n* = 77)
Wound discharge	50	31	4	85 (1.3)	*Pseudomonas* spp. (*n* = 20)	*S. aureus* (*n* = 18)	*Enterococcus* spp. (*n* = 9)
Biological fluid	53	19	3	75 (1.1)	*Pseudomonas* spp. (*n* = 16)	*Klebsiella* spp. (*n* = 11)	*Acinetobacter* spp. (*n* = 9)
Bronchoalveolar lavage	11	2	1	14 (0.2)	*Pseudomonas* spp. (*n* = 3)	*S. maltophilia* (*n* = 2)	*Citrobacter* spp. (*n* = 2)
Feces	1	0	0	1 (0.015)	*Salmonella* spp. (*n* = 1)	−	−

^a^ Total of isolates in each group, and percentage among all isolates. ^b^ Microbial genus or species was identified by phenotypic methods using conventional microbiological methods and automated identification systems (VITEK^®^ 2 and/or BD Phoenix™); ^c^ The tissue category comprised skin specimens, soft tissue specimens, and debridement tissue; *n*: number of isolates; GNB: Gram-negative bacteria; GPB: Gram-positive bacteria; CoNS: Coagulase-negative staphylococci; *S. aureus*: *Staphylococcus aureus*; *S. maltophilia*: *Stenotrophomonas maltophilia*; −: not detected.

**Table 3:** Temporal trends in relative abundance of major microbiological groups in a burn unit (2014–2024).

Group	Total ^a^	IRR/Year ^b^	95% CI Low	95% CI High	*p*–Value ^c^
Gram-negative Bacteria	4103 (60.6)	0.998	0.992	1.004	0.426
Gram-positive Bacteria	1957 (28.9)	1028	1.010	1.045	0.0018
Fungi	709 (10.5)	0.942	0.906	0.979	0.0027

^a^ Total of isolates in each group, and percentage among all isolates. ^b^ Trends were assessed using count regression models and are presented as incidence rate ratios (IRRs) per year with 95% confidence intervals (95% CIs). ^c^ Statistical significance was determined using false discovery rate (FDR)-adjusted *p*-values.

**Table 4:** Temporal trends in relative abundance of major microbiological taxa in a burn unit (2014–2024).

Microorganism ^a^	Total ^b^	IRR/Year ^c^	95% CI Low	95% CI High	*p*–Value ^d^	*q*-Value ^d^
*Acinetobacter* spp.	1172 (17.3)	0.973	0.956	0.991	0.0028	0.0084
CoNS	927 (13.7)	1.009	0.986	1.032	0.453	0.473
*Pseudomonas* spp.	856 (12.6)	1.025	0.999	1.051	0.0581	0.0968
*Klebsiella* spp.	727 (10.7)	0.988	0.962	1.014	0.368	0.459
*Candida* spp.	515 (7.6)	0.923	0.874	0.974	0.0034	0.0084
*Staphylococcus aureus*	460 (6.8)	1.053	1.025	1.082	1.6 × 10^−4^	8.0 × 10^−4^
*Enterococcus* spp.	396 (5.9)	1.076	1.021	1.135	0.0067	0.0133
*Enterobacter* spp.	326 (4.8)	0.931	0.904	0.958	1.4 × 10^−6^	1.4 × 10^−5^
*Serratia* spp.	258 (3.8)	1.050	0.972	1.135	0.215	0.307
*Escherichia coli*	163 (2.4)	0.981	0.931	1.034	0.473	0.473

^a^ Microbial genus or species was identified by phenotypic methods using conventional microbiological techniques and automated identification systems (VITEK^®^ 2 and/or BD Phoenix™); ^b^ Total of isolates in each group, and percentage among all isolates. ^c^ Trends were assessed using count regression models and are presented as incidence rate ratios (IRRs) per year with 95% confidence intervals (95% CIs). ^d^ Statistical significance was determined using false discovery rate (FDR)-adjusted *p*-values. CoNS: Coagulase-negative staphylococci.

## Data Availability

The original contributions presented in this study are included in this article/Supplementary Materials. Further inquiries can be directed to the corresponding author.

## Supplementary Materials

The following supporting information can be downloaded at https://www.mdpi.com/article/10.3390/epidemiologia7050130/s1, Figure S1: Pareto chart of microbiological isolates recovered from burn patients at a tertiary care hospital in southern Brazil (2014–2024). Pareto charts were generated to summarize the relative contribution of microbial taxa to the total number of isolates. Statistical analyses were performed using R software (version 4.3.0) and IBM SPSS Statistics (version 26.0).; Figure S2: Distribution of microbiological groups (Gram-negative bacteria, Gram-positive bacteria, and fungi) according to clinical specimen type.

## Abbreviations

The following abbreviations are used in this manuscript:

BICU	Burn intensive care unit
CI	Confidence interval
CoNS	Coagulase-negative staphylococci
FDR	False discovery rate
GNB	Gram-negative bacteria
GPB	Gram-positive bacteria
HAIs	Healthcare-associated infections
IRRs	Incidence rate ratios
WHO	World Health Organization
