# Supplementary material for: Temporal Trends and Epidemiological Patterns of Clinically Relevant Microorganisms in a Burn Unit: An 11-Year Retrospective Study from a Tertiary Hospital in Southern Brazil
Source: Epidemiologia (Basel). 2026 Sep 17;7(5):130. doi: 10.3390/epidemiologia7050130 (PMC13600085; doi:10.3390/epidemiologia7050130)
Supplement: Supplementary file 1 [file epidemiologia-07-00130-s001.zip › epidemiologia-4480673-supplementary.pdf]

Article

Temporal Trends and Epidemiological Patterns of Clinically Relevant Microorganisms in a Burn Unit: An 11-Year Retrospective Study from a Tertiary Hospital in Southern Brazil

Raquel Lima Palermo [1], Isabela Madeira de Castro [1], Lais Fernanda de Almeida Spoladori [1], Eliandro Reis Tavares [1], Marcia Regina Eches Perugini [2], Lucy Megumi Yamauchi [1], Eliana Carolina Vespero [2,*] and Sueli Fumie Yamada-Ogatta [1,2,*]

1 Programa de Pós-graduação em Microbiologia, Departamento de Microbiologia, Universidade Estadual de Londrina, Londrina CEP 86057-970, Brazil; raquellima.palermo@gmail.com (R.L.P.); isabela.mcastro@uel.br (I.M.d.C.); lais.spoladori@gmail.com (L.F.d.A.S.); tavares.eliandro@uel.br (E.R.T.); lionilmy@uel.br (L.M.Y.)

2 Programa de Pós-graduação em Fisiopatologia Clínica e Laboratorial, Departamento de Patologia, Análises Clínicas e Toxicológicas, Universidade Estadual de Londrina, Londrina CEP 86038-350, Brazil; marciaperugini@uel.br (M.R.E.P.)

* Correspondence: elianacv@uel.br; Tel.: +55-(43)-3371-2200; ogatta@uel.br; Tel.: +55-(43)-3371-5503

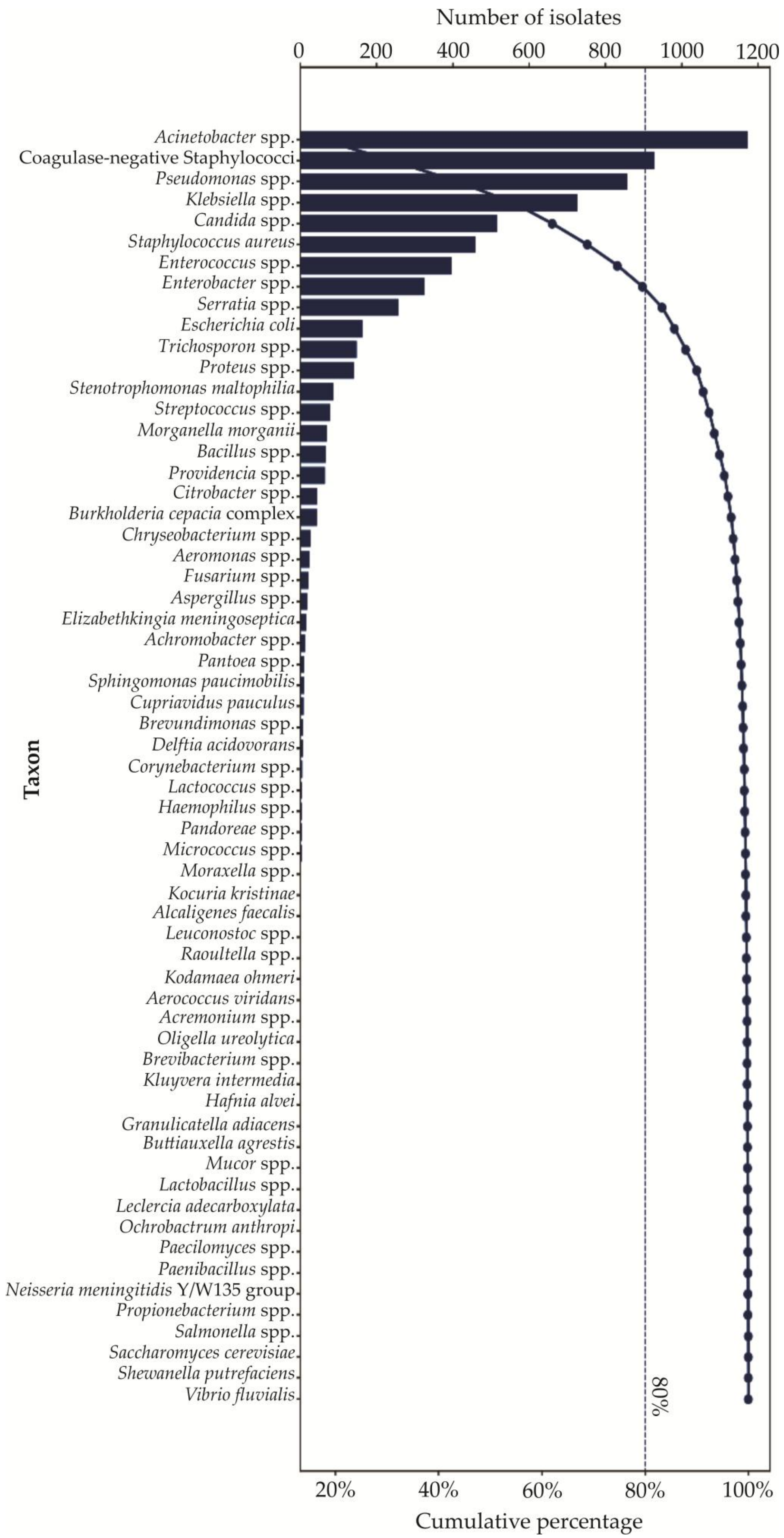
Number of isolates
0
200
400
600
800
1000
1200
Taxon
Acinetobacter spp.
Coagulase-negative Staphylococci
Pseudomonas spp.
Klebsiella spp.
Candida spp.
Staphylococcus aureus
Enterococcus spp.
Enterobacter spp.
Serratia spp.
Escherichia coli
Trichosporon spp.
Proteus spp.
Stenotrophomonas maltophilia
Streptococcus spp.
Morganella morganii
Bacillus spp.
Providencia spp.
Citrobacter spp.
Burkholderia cepacia complex
Chryseobacterium spp.
Aeromonas spp.
Fusarium spp.
Aspergillus spp.
Elizabethkingia meningoseptica
Achromobacter spp.
Pantoea spp.
Sphingomonas paucimobilis
Cupriavidus pauculus
Brevundimonas spp.
Delftia acidovorans
Corynebacterium spp.
Lactococcus spp.
Haemophilus spp.
Pandoreae spp.
Micrococcus spp.
Moraxella spp.
Kocuria kristinae
Alcaligenes faecalis
Leuconostoc spp.
Raoultella spp.
Kodamaea ohmeri
Aerococcus viridans
Acremonium spp.
Oligella ureolytica
Brevibacterium spp.
Kluyvera intermedia
Hafnia alvei
Granulicatella adiacens
Buttiauxella agrestis
Mucor spp.
Lactobacillus spp.
Leclercia adecarboxylata
Ochrobactrum anthropi
Paecilomyces spp.
Paenibacillus spp.
Neisseria meningitidis Y/W135 group
Propionebacterium spp.
Salmonella spp.
Saccharomyces cerevisiae
Shewanella putrefaciens
Vibrio fluvialis
80%
20%
40%
60%
80%
100%
Cumulative percentage

Figure S1. Pareto chart of microbiological isolates recovered from burn patients at a tertiary care hospital in southern Brazil (2014–2024). Pareto charts were generated to summarize the relative contribution of microbial taxa to the total number of isolates. Statistical analyses were performed using R software (version 4.3.0) and IBM SPSS Statistics (version 26.0).

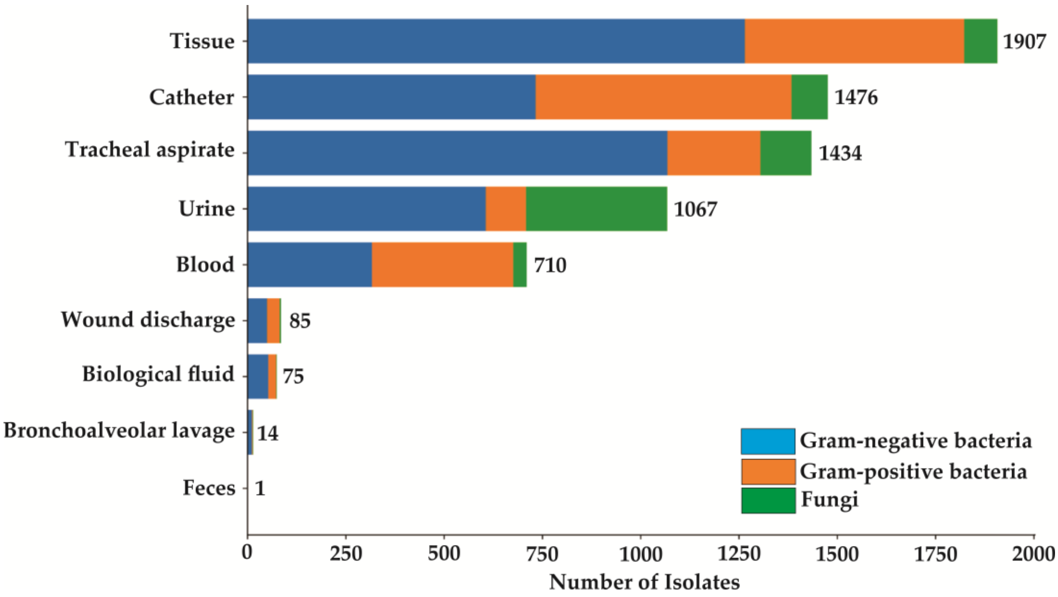

Figure S2. Distribution of microbiological groups (Gram-negative bacteria, Gram-positive bacteria, and fungi) according to clinical specimen type.
